# Variation in *Wolbachia* effects on *Aedes* mosquitoes as a determinant of invasiveness and vectorial capacity

**DOI:** 10.1038/s41467-018-03981-8

**Published:** 2018-04-16

**Authors:** Jessica G. King, Caetano Souto-Maior, Larissa M. Sartori, Rafael Maciel-de-Freitas, M. Gabriela M. Gomes

**Affiliations:** 1 CIBIO-InBIO, Centro de Investigação em Biodiversidade e Recursos Genéticos, Universidade do Porto, Vairão, 4485-661 Portugal; 2 Instituto Gulbenkian de Ciência, Oeiras, 2780-156 Portugal; 3 Instituto de Matemática e Estatística, Universidade de São Paulo, São Paulo, 05508-090 Brazil; 4 Laboratório de Transmissores de Hematozoários, IOC, Fundação Oswaldo Cruz, Rio de Janeiro, 21040-900 Brazil; 5 Liverpool School of Tropical Medicine, Liverpool, L3 5QA UK; 6 Present Address: Institute of Evolutionary Biology, School of Biological Sciences, University of Edinburgh, Edinburgh, EH9 3FL UK

## Abstract

*Wolbachia* has been introduced into *Aedes aegypti* mosquitoes to control the spread of arboviruses, such as dengue, chikungunya and Zika. Studies showed that certain *Wolbachia* strains (such as *w*Mel) reduce replication of dengue viruses in the laboratory, prompting the release of mosquitoes carrying the bacterium into the field, where vectorial capacity can be realistically assessed in relation to native non-carriers. Here we apply a new analysis to two published datasets, and show that *w*Mel increases the mean and the variance in *Ae. aegypti* susceptibility to dengue infection when introgressed into Brazil and Vietnam genetic backgrounds. In the absence of other processes, higher mean susceptibility should lead to enhanced viral transmission. The increase in variance, however, widens the basis for selection imposed by unexplored natural forces, retaining the potential for reducing transmission overall.

## Abstract (web summary)

*Wolbachia* infection in mosquitoes reduces dengue virus spread under specific lab conditions, prompting its use in disease control. Here, King et al. show that *Wolbachia* increases mean and variance in mosquito susceptibility and explain how this affects *Wolbachia* invasion and dengue transmission.

## Introduction

*Aedes* mosquitoes are competent vectors for several viral diseases of humans. *Wolbachia* is a symbiont bacterium of arthropods^1^, shown to manipulate the reproduction of its hosts to facilitate invasion of carriers, and to modify host responses to viral infections, notably in *Ae. aegypti*^2,3^, indicating a potential role in the control of viral diseases^4,5^.

*Wolbachia* is commonly transmitted vertically from mother to offspring. Horizontal transmission between host populations or species is rare, but when successful the spread of the symbiont may be facilitated by multiple mechanisms. First, there are specific forms of reproductive manipulation, such as cytoplasmic incompatibility between sperm of male carriers and eggs of female non-carriers, which result in unviable crosses, suppressing non-carrier populations and, consequently, conferring competitive advantage to *Wolbachia* carriers^6^. Second, *Wolbachia* has been shown to confer a degree of protection against viral pathogens^7,8^, which facilitates its establishment in host populations when such pathogens are present^9^. Third, substantial inter-individual variation in this effect has been found^10^, which further increases *Wolbachia* invasiveness through a process of cohort selection^11^. Cohort selection (also known as survival bias) occurs whenever there is variation and selection, irrespective of whether the trait under study is heritable. In the case of susceptibility to infection (i.e., probability of infection per unit of pathogen challenge), individuals with higher susceptibility are more likely to be affected and therefore tend to be removed from the population at risk sooner than those with lower susceptibility, leading to a decrease in mean susceptibility among survivors as a cohort ages. Thus, by merely increasing variation in host susceptibility to pathogens, *Wolbachia* increases the resilience of its carrier populations and facilitates its own invasion.

Due to the second and third mechanisms above, *Wolbachia* invasion implies the replacement of a host population by another whose susceptibility to the pathogen under study is lower. These findings support the notion that *Wolbachia* carriage by insect vectors of human pathogens, such as mosquitoes, might be manipulated to reduce disease transmission^5,12^.

The mosquito *Ae. aegypti*, primary vector of dengue, is not a natural host of *Wolbachia*. Transinfection of various strains has been performed in the laboratory and shown to reduce vector competence, on average, when mosquitoes were challenged with high viral doses. This has led to field releases of *w*Mel-transinfected *Ae. aegypti* in 10 countries—Australia, Brazil, Colombia, Indonesia, Sri Lanka, India, Vietnam, Kiribati, Fiji and Vanuatu—to evaluate its effectiveness in reducing dengue and other mosquito-borne diseases in human populations^13^.

Here we explore the effects of *Wolbachia* on the susceptibility distributions of two *Ae. aegypti* populations—Rio de Janeiro, Brazil, and Ho Chi Minh City, Vietnam—to dengue viruses. The wide variability in exposure doses naturally experienced by mosquitoes prompted the adoption of dose-response experimental designs^4,14^ for the inference of distributions of *Wolbachia* effects, which were then inserted in high-dimensional mathematical models to investigate the conditions for *Wolbachia* invasion and its impact on dengue transmission. We find that the symbiont increases the mean and the variance in *Ae. aegypti* susceptibility to dengue infection. While higher mean susceptibility alone should lead to enhanced viral transmission, the increase in variance widens the basis for selection imposed by unexplored natural forces, such as mosquito pathogens, which need to be catalogued before net effects can be predicted.

## Results

### Susceptibility distributions of mosquitoes to dengue viruses

**Fig. 1:**
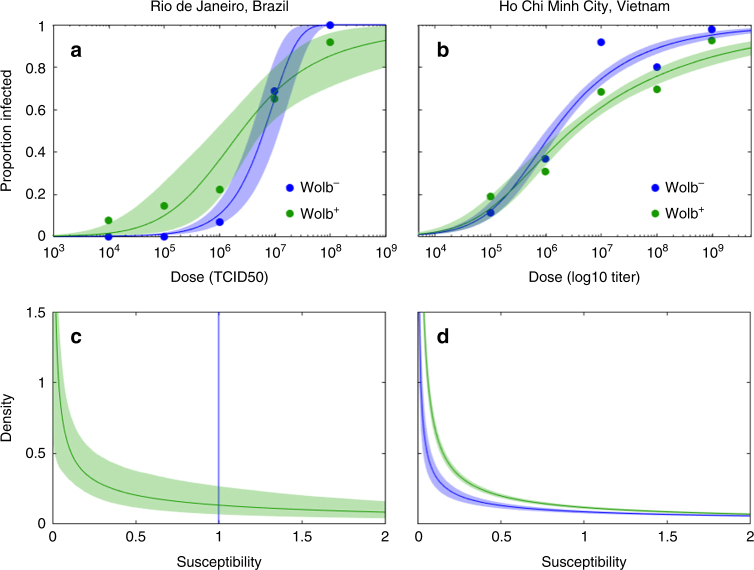
Susceptibility distributions estimated from dose-response curves. **a**, **b** Dose-response curves for *Wolbachia*-free (Wolb^−^, in blue) and Wolbachia-carrier (Wolb^+^, in green) populations given data (dots) collected from Rio de Janeiro, Brazil (**a**) and Ho Chi Minh City, Vietnam (**b**). **c**, **d** Susceptibility distributions estimated by fitting dose-response models to the data (**c** Brazil; **d** Vietnam) as described in Methods section. Estimated shape (*α*) and scale (*θ*) parameters of the gamma distributions (maximum a posteriori probability, median and 95% credible intervals) are provided in Table 1. Highest probability moments: **c**, mean(Wolb^−^) = 1; variance(Wolb^−^) = 0; mean(Wolb^+^) = 6.920; variance(Wolb^+^) = 143.7; **d**, mean(Wolb^−^) = 1; variance(Wolb^−^) = 2.776; mean(Wolb^+^) = 1.490; variance(Wolb^+^) = 10.85

Figure 1 shows the estimation of susceptibility distributions for populations of *Ae. aegypti* (Wolb^−^ denoting non-carriers of *Wolbachia*, and Wolb^+^ the carriers). Rio de Janeiro mosquitoes were challenged by injection with dilutions of a serotype 1 virus previously isolated from a patient and amplified, whereas in Ho Chi Minh City the adopted procedure was feeding on viremic blood from multiple infected patients. Either case provided suitable data for fitting dose-response models^15,16^ as described in Methods section. Figure 1a, b shows the data and model fittings, which resulted in the susceptibility distributions plotted in Fig. 1c, d. Models with gamma-distributed susceptibilities performed better than their homogeneous counterparts, according to model selection criteria, except in the case of Brazilian Wolb^−^ challenged by injection. Estimated parameters are shown in Table 1 and model selection was performed by deviance information criterion (DIC) (Supplementary Tables 1 and 2). *Wolbachia* consistently increased the mean susceptibility of mosquitoes to dengue viruses (by average factors of 6.9 and 1.5 in the experiments of Brazil and Vietnam, respectively), and in addition it increased the variance-to-mean ratio in the trait (to 20.83 in Brazil, and 7.2 in Vietnam). The remainder of this paper addresses the implications of these findings on the prospects for *Wolbachia* to invade *Ae. aegypti* populations and eliminate dengue as a human disease.

**Table 1:** Parameter estimates from dose-response model fitting to experimental data

Parameter	*p*	${\mathbf{\alpha}} _{{\bf Wolb}^{\bf +}}$	${\mathbf{\theta}} _{{\bf Wolb}^{\bf +}}$
**Brazil**			
MAP	1.149 × 10^−7^	0.3332	20.77
Median	1.066 × 10^−7^	0.3465	26.54
95% CI	5.720 × 10^−8^	0.1547	2.566
	1.837 × 10^−7^	0.8907	447.0

Parameter	${\mathbf{\alpha}} _{{\bf Wolb}^{\bf -}}$	${\mathbf{\theta}} _{{\bf Wolb}^{\bf -}}$	${\mathbf{\alpha}} _{{\bf Wolb}^{\bf +}}$	${\mathbf{\theta}} _{{\bf Wolb}^{\bf +}}$
**Vietnam**				
MAP	0.3602	347.1	0.2047	910.3
Median	0.3652	401.9	0.2064	944.5
95% CI	0.3045	235.4	0.1677	437.7
	0.4366	761.4	0.2522	2363

MAP denotes maximum a posteriori probability. In Brazil, *p* is the probability of infection for a Wolb^−^ mosquito per unit of viral challenge (TCID_50_), and ${\rm \alpha}_{{\rm Wolb}^+}$, $\theta_{{\rm Wolb}^+}$ are the shape and scale parameters, respectively, of the gamma distribution determining the susceptibility factors of Wolb^+^ mosquitoes in relation to Wolb^−^ (i.e., with respect to *p*). In Vietnam *p*_aux_ = 10^−8^ (per log_10_ viral titre (RNA copies/ml)), was used as an auxiliary parameter, while ${\rm \alpha}_{{\rm Wolb}^--}$, $\theta _{{\rm Wolb^-}}$ and ${\rm \alpha}_{{\rm Wolb}^+}$, $\theta _{{\rm Wolb^+}}$ define the distributions of susceptibility of Wolb^−^ and Wolb^+^ mosquitoes, respectively, in relation to *p*_aux_

### *Wolbachia* invasion dynamics

The first reports of *Wolbachia* interfering with host susceptibility to pathogens are dated no more than a decade ago^7,8^, and association studies suggest that general mechanisms modify susceptibility to broad spectra of viruses to similar extents^17^. The advent of sequence-independent techniques, based on small RNAs produced by the host, to characterize insect viruses^18^, opens exciting opportunities to study the depth of such associations. In anticipation, a suite of unspecified pathogens capable to infect mosquitoes is contemplated in our models by a parameter *λ*_M_, whose value can be set to any positive real number or zero, and individual susceptibility to the pathogen ensemble is treated as being perfectly correlated with susceptibility to dengue viruses. Moreover, these pathogens are assumed to be host generalists^11^ (i.e., capable to infect other host species), and thus their abundance is not determined by the specific interactions with *Ae. aegypti*. This enables an initial exploration of the expected impacts of *Wolbachia*-induced variation in mosquito susceptibility to viruses, which may be refined if richer pathogen-specific datasets become available. In a similar vein, possible correlations or trade-offs between viral susceptibility and other life-history traits are not contemplated at this stage.

**Fig. 2:**
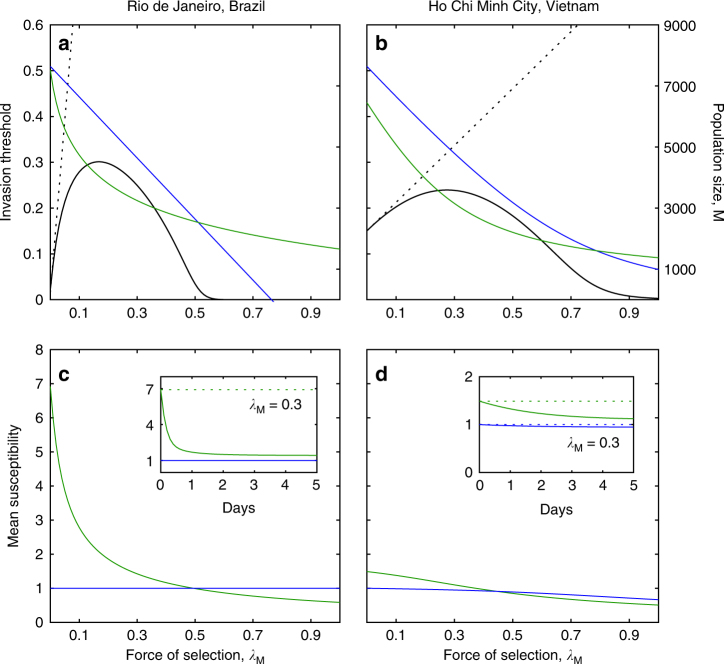
Threshold for *Wolbachia* invasion and cohort selection on susceptibility. **a**, **b** Invasion threshold for *Wolbachia* carriers over a range of forces of selection by mosquito pathogens, obtained by introducing the susceptibility distributions estimated in Fig. 1 in a population dynamic model described in Methods section (solid back curves). Dotted lines show the results of the same calculation if only the means had been used to describe the susceptibility of each population. Other model outputs are the population sizes of *Wolbachia*-free (Wolb^−^, in blue) and *Wolbachia*-carrier (Wolb^+^, in green) populations at fixation, in Rio de Janeiro, Brazil (**a**) and Ho Chi Minh City, Vietnam (**b**). **c**, **d** Effects of cohort selection on the mean susceptibility of each population once equilibrium has been reached under a range of selection forces. The insets illustrate how mean susceptibilities develop over time, from the moment populations are released until equilibria are established, under one specific force of selection (*λ*_M_ = 0.3). The changes in mean susceptibility depicted in the insets are accompanied by reductions in variance. Variance-to-mean ratios in Wolb^+^ populations changed from 20.83 to 10.10 in Brazil (**c**), and from 7.200 to 0.7715 in Vietnam (**d**)

Figure 2 addresses the invasiveness of mosquito populations carrying *Wolbachia*, upon releases in Rio de Janeiro and Ho Chi Minh City, accounting for the susceptibility distributions estimated here in conjunction with various demographic parameters estimated previously (Table 2), as a function of mosquito pathogen abundance, *λ*_M_. The solid black curve in Fig. 2a, b depicts the threshold frequency that *Wolbachia* carriers must attain in the population for their invasion to be predicted^19^, despite specific costs associated with the symbiosis, such as measured reductions in fecundity and lifespan^20–22^. Population densities where *Wolbachia* is absent or fixed are also shown for completeness. The figure reveals how mosquito pathogens, which act as a force of selection upon susceptibility distributions, are expected to facilitate *Wolbachia* invasions due to the higher variance in the trait among carriers.

**Table 2:** Parameters for *Wolbachia* invasion and dengue transmission models

	**Symbol, definition**	**Value**	**Reference**
Mosquito population dynamics	*a*, birth rate	0.789 day^−1^	Dutra et al.^20^
	*b*, density-independent death rate	0.025 day^−1^	Dutra et al.^20^
	*k*, density-dependent death rate	0.0001 (insect × day) ^−1^	This study
	*s*_h_, proportion of unviable offspring in incompatible crosses	0.995 (BR); 1 (VN)	Dutra et al.^20^ ; Joubert et al.^22^
	*s*_f_, relative fecundity reduction in *Wolbachia*-carriers	0 (BR); 0.15 (VN)	Dutra et al.^20^ ; Nguyen et al.^21^
	*s*_l_, relative longevity reduction in *Wolbachia*-carriers	0.33 (BR); 0.012 (VN)	Dutra et al.^20^ ; Joubert et al.^22^
	*λ*_M_, force of selection by mosquito pathogens	0–2 day^−1^	This study
Human population dynamics	*μ*, birth and death rate, given life expectancy ≈ 75 years	1/(365 × 75) day^−1^	WHO, Global Health Observatory, Life Expectancy (2015)^43^
	*γ*, rate of recovery from dengue infection	1/7 day^−1^	Sabin (1952)^44^
	*φ*, rate of loss-of-immunity	2/365 day^−1^	Sabin (1952)^44^
Transmission dynamics	*ρ*_U_, proportion of infected *Wolbachia*-free mosquitoes who are infectious	0.6046 (mean); 0.4862 (min.); 0.7407 (max.)	Ferguson et al.^4^
	*ρ*_W_, proportion of infected *Wolbachia*-carrying mosquitoes who are infectious	0.2729 (mean); 0.2102 (min.); 0.3160 (max.)	Ferguson et al.^4^
	*β*, human-to-mosquito transmission rate	0–2.5 day^−1^	This study

The dotted lines in Fig. 2a, b shows how the invasion threshold would be expected to increase with exposure to mosquito pathogens given the increases in mean susceptibility attributed to *Wolbachia* in our analysis of experimental data, if the changes in variance had been ignored. When entire distributions are considered, the greater variance among carriers reverses the trend and, if pathogen exposure is sufficiently high, the threshold may eventually vanish. This is due to cohort selection, which is disabled in mean field approximations. When a population with a given susceptibility distribution is released from the laboratory to some pathogen-richer environment, pathogen pressure determines the profile that will be effectively established. Since pathogens affect predominantly those individuals who are more susceptible, mean susceptibility decays with time since release (Fig. 2c, d), facilitating invasion. Everything else being the same, invasion thresholds are lower for populations with higher variance. In this process, variance-to-mean ratios also decrease, which is consistent with the notion that the lower variances exhibited by resident (compared to introduced) mosquitoes are a natural consequence of previous exposure to selection.

**Fig. 3:**
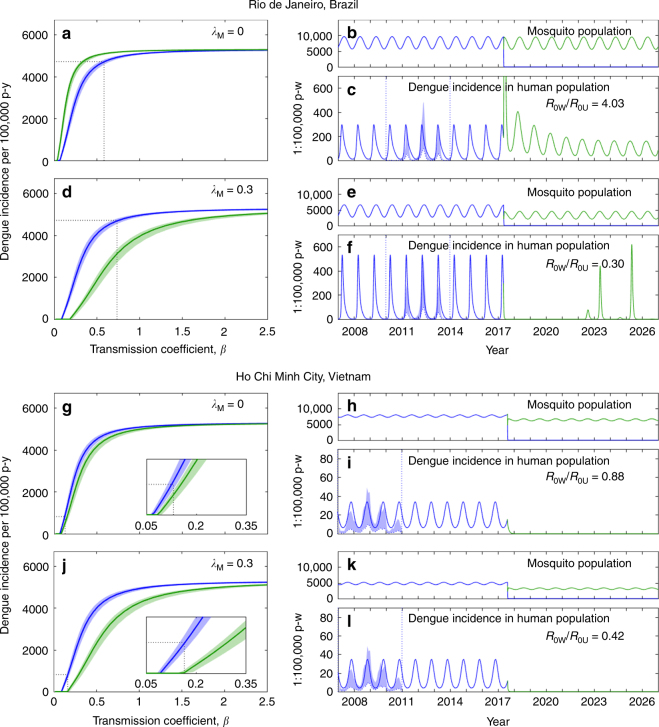
Projected impact of *Wolbachia* on dengue transmission in human populations. Colours indicate whether transmission is by *Wolbachia*-free (Wolb^−^, in blue) or *Wolbachia*-carrier (Wolb^+^, in green) mosquitoes. Left panels show dengue incidence versus transmission coefficient plotted from equilibrium solutions of a 4-serytope dengue model (Methods section) on human populations with heterogeneous risk (variance-to-mean ratio of 4): **a**, **d** mosquitoes parameterized from experiments in Brazil and released under different forces of selection by mosquito pathogens: **a** *λ*_M_ = 0; **d** *λ*_M_ = 0.3. **g**, **j** same as **a**, **d**, for Vietnam. Equilibrium curves adopted mean proportions of infected mosquitoes with detectable virus in the salivary glands^3^, and shaded areas represent lower and upper bounds encountered when mosquitoes were stratified by challenge dose. Dotted lines mark dengue incidence in Rio de Janeiro, and Ho Chi Minh City, averaged over a 4-year period and scaled by an expansion factor of 5. Right panels show model simulations of *Wolbachia* releases taking place in 2017, starting from the conditions on the respective left panels: **b**, **e**, **h**, **k** mosquito population sizes; **c**, **f**, **i**, **l** dengue epidemics in humans (shaded areas refer to notification data; lower bound showing the original data and upper bound the data multiplied by an expansion factor of 5). Parameter values: *β* = 0.59 (**c**), *β* = 0.74 (**f**), *β* = 0.13 (**i**), *β* = 0.17 (**l**); *A* = 0.25, *Β* = 0.25 (**b**, **c**, **e**, **f**); *A* = 0.05, *Β* = 0.1 (**h**,** i**, **k**, **l**)

The earlier releases of *Ae. aegypti* mosquitoes transinfected with *w*Mel occurred in Northern Queensland, Australia—first in isolated communities (Gordonvale and Yorkeys Knob, 2011)^12^, and later in a city (Cairns, 2013)^23^—where ongoing monitoring provides growing datasets to test models. In these settings, the invasion threshold was estimated to be in the range 0.2–0.35. According to our Fig. 2a, b, thresholds in this range are expected for Rio de Janeiro and Ho Chi Minh City Vietnam when pathogen pressure is included (*λ*_M_ around 0.3), but would appear too high otherwise (that is, if *λ*_M_ = 0). Also notable is the lower amplitude of seasonal fluctuations observed in *Wolbachia*-carrying populations (relative to non-carriers) within release zones^23^, which would be predicted by cohort selection on a population with higher variance in susceptibility (Fig. 3e, k).

### Dengue transmission dynamics

Figure 3 shows the results of further incrementing the model with dengue transmission in a human population^24^, calibrating the outputs prior to *Wolbachia* releases to dengue notification data in the two study cities, and then simulating releases in 2017^25,26^. A 4-serotype model was necessary to meet the higher incidences observed in Brazil (Supplementary Fig. 1). In Rio de Janeiro, where *Wolbachia* was estimated to increase mean susceptibility by a factor of 6.9, dengue transmission would be slightly increased by *Wolbachia* fixation in the absence of selection (Fig. 3a). However, when selection by pathogens is present, as expected, a population of *Wolbachia*-carriers with the same characteristic can reduce dengue transmission substantially, by an extent that increases with the intensity of pathogen exposure. In Ho Chi Minh City, the predictions are less extreme because *Wolbachia*-mediated increases in the mean and variance of the susceptibility distribution are smaller. In the absence of pathogens, we expect a slight reduction in dengue transmission (Fig. 3g), despite the increase in mosquito mean susceptibility to infection to a factor of 1.5. This is due to a reduction in infectivity (*ρ*_W_/*ρ*_U_ = 0.45), inferred as the proportion of infected mosquitoes that had virus in the salivary glands^4^. The same factor was adopted for the Brazil simulations, although shown by itself insufficient to reduce transmission in that setting. The effects of *Wolbachia* on the reproduction number, *R*_0_, were calculated for each scenario, resulting in factors in the ranges 0.30–4.0 for Brazil and 0.42–0.88 for Vietnam, with the amplitude of the range attributed to the intensity of selection imposed by mosquito pathogens.

The results in Fig. 3 were obtained considering a human population with heterogeneous risk of acquiring and transmitting dengue infection. Little is known about risk distributions for acquiring dengue in humans, although estimates for other diseases indicate variance-to-mean ratios in the 4–20 range^27,28^. Here we have adopted the conservative value of 4 and show, for comparison, the corresponding results generated under variance-to-mean ratios of 20 (Supplementary Fig. 2) and 0 (Supplementary Fig. 3). Reductions in dengue transmission appear greater under more homogeneous human population models, although this would not have been captured by *R*_0_ ratios alone. In Vietnam, elimination of dengue is predicted under either sufficiently low heterogeneity in the human population, or sufficiently high-cohort selection in the mosquito population, whereas in Brazil, where baseline incidence is higher, stable elimination is not predicted in any of the scenarios contemplated here.

## Discussion

Analysis of two independently generated sets of dose-infectivity curves, shows that *Wolbachia* consistently increases the mean and the variance in *Ae. aegypti* mosquito susceptibility to dengue viruses. These effects appear greater in Brazil, where mosquitoes were challenged by injection^14^, than in Vietnam, where challenges were by ingestion of viremic blood from infected patients^4^. The finding that *Wolbachia* increases variation in susceptibility to a virus is compatible with a study previously carried out in *Drosophila melanogaster* flies^10^ and demands devoted research.

*Wolbachia* has often been found to protect hosts against pathogens, but not always. There is a cumulative number of studies, both in natural and artificial *Wolbachia*-host systems, which report that *Wolbachia* enhances^29,30^ or has no noticeable effect^31,32^ on specific pathogens. To what extent these discrepancies are real or methodological is to be inquired. In the light of our findings, a more comprehensive analysis of all available data is warranted. First, we convey that no conclusions can be drawn from standard single-dose experimental challenge designs. Second, we demonstrate that inclusive dose-response analyses may lead to results which contradict those obtained by averaging single-dose findings. Third, we find that by consistently increasing variation in host susceptibility to viruses, *Wolbachia* transinfection creates a population whose effective mean susceptibility is highly sensitive to natural selective pressures operating in specific release sites. Given that *Wolbachia* appears to modify host susceptibility to a broad spectrum of pathogens^7,17^, reliable predictions of invasiveness and vectorial capacity of transinfected mosquitoes require an informed account of natural mosquito pathogens and their interplay with *Wolbachia*.

Having exposed the fundamental roles of individual variation in host (mosquito or human) susceptibility, or exposure, to infections in leveraging population measures, the adoption of homogeneous models, for assessing and predicting the response to interventions, is no longer an option. A dose-response experimental design analogous to that performed here has recently been adopted to assess a vaccine against a virus of rainbow trout^33^, while suitable study designs on natural endemicity gradients^34^ can enable the estimation of essential heterogeneity parameters from field settings^27,35,36^, just like dose-response experimental designs do in the laboratory.

## Methods

### Data preparation

We reanalyse two previously published datasets^4,14^, where *Aedes aegypti* mosquitoes were challenged with doses of dengue virus spanning a suitable range, and viral titres were subsequently measured to determine infection status. The experimental challenge procedures were performed on two mosquito populations: carrying a *w*Mel *Wolbachia* strain (Wolb^+^); and not carrying any *Wolbachia* (Wolb^−^). In one of the experiments^14^, mosquitoes from Rio de Janeiro, Brazil, were challenged by injection, while in the other^4^, mosquitoes from Ho Chi Minh City, Vietnam, were challenged by feeding on viremic blood from infected patients. The proportions of mosquitoes with detectable virus were assessed at various days after infection (range 3–14 in Brazil; and 7–18 in Vietnam), and data were formatted in a dose-response manner, with dose referring to challenge and response representing probability of infection.

### Dose-response model

Adopting established formulations^15,16^, we denote by *d* the viral challenge dose (measured in units of 50% tissue culture infective dose [TCID_50_] in Brazil, and log_10_ viral titre [RNA copies/ml] in Vietnam) and *p* a measure of infectivity to a mosquito host per unit of viral challenge; the number of infecting units per host is assumed to follow a Poisson distribution with mean *pd*. If all hosts are equally susceptible, the probability of a host remaining uninfected after viral challenge is the zero term of the distribution, leading to a probability of infection $r_{{\rm hom}} = 1 - e^{ - pd}$. When individual hosts vary in their susceptibility to infection, we consider the infectivity of each unit of challenge to vary between hosts according to a gamma distribution $q\left( x \right) = \frac{{x^{\alpha - 1}e^{ - x/\theta }}}{{{\mathrm{\Gamma }}(\alpha )\theta ^\alpha }}$, where *α* and *θ* are the shape and scale parameters, respectively, resulting in the modified dose-response model $r_{{\rm het}} = 1 - \mathop {\int }\limits_0^\infty e^{ - xpd}q\left( x \right){\rm d}x$, or its closed form $r_{{\rm het}} = 1 - \left[ {1/\left( {1 + \theta pd} \right)} \right]^\alpha$, which is obtained by Laplace transform. The factors *x* are dimensionless measures of relative susceptibility between hosts.

### Susceptibility distribution estimation

Dose-response models are fitted to the experimental data using a Bayesian Markov Chain Monte Carlo (MCMC) based method^10^, implemented in Python programming language with PyMC package^37^, assuming uniform priors, to estimate distributions of susceptibility of mosquito populations to dengue viruses. The likelihood of the parameters given the data is computed using binomial distributions written for a given dose as

1 $$P\left( {k{\mathrm{|}}N,d,p} \right) = \left( {\begin{array}{*{20}{c}} N \\ k \end{array}} \right)r^k\left( {1 - r} \right)^{N - k},$$

where *N* is the number of mosquitoes being challenged, *r* is the probability of obtaining a successful response from each challenge (i.e., successful infection by the virus), which is a function of *p* and *d*, according to either homogeneous ($r_{{\rm hom}}$) or heterogeneous ($r_{{\rm het}}$) model formulations given above, and *k* is the observed number of infected mosquitoes (i.e., with detectable viral titres).

The fit of the homogeneous model to data across *D* doses gives an estimate of parameter *p*, whose log-likelihood is given by a sum of binomial distributions

2 $$P\left( {p|\vec k,\vec N,\vec d} \right) = \mathop {\sum }\limits_{i = 1}^D {\rm log}\left( {\left( {\begin{array}{*{20}{c}} {N_i} \\ {k_i} \end{array}} \right)r_i^{k_i}\left( {1 - r_i} \right)^{N_i - k_i}} \right),$$

where $\vec k$, $\vec N$ and $\vec d$ are vectors composed of $k_i$, $N_i$ and $d_i$, respectively, and $r_i = 1 - e^{ - pd_i}$. For fitting the heterogeneous model, the log-likelihood is given by

3 $$P\left( {\alpha ,\theta |\vec k,\vec N,\vec d,p} \right) = \mathop {\sum }\limits_{i = 1}^D {\rm log}\left( {\left( {\begin{array}{*{20}{c}} {N_i} \\ {k_i} \end{array}} \right)r_i^{{\,}{k_i}}\left( {1 - r_i} \right)^{N_i - k_i}} \right),$$

where $r_i = 1 - \left[ {1/\left( {1 + \theta p_{{\rm aux}}d_i} \right)} \right]^\alpha$, and $p_{{\rm aux}}$ is treated as a fixed auxiliary parameter.

Brazilian and Vietnamese studies are analysed separately. Model formulations where the susceptibility of individual mosquitoes is considered homogeneous or gamma-distributed are both fitted to Wolb^−^ data. The deviance information criterion (DIC) is applied to select between the two formulations, favouring homogeneous in Brazil, and heterogeneous in Vietnam (adopting *p*_aux_ = 1 in Supplementary Table 1, and *p*_aux_ = 10^−8^ in Supplementary Table 2).

The selected model is then extended with a gamma-distributed susceptibility for Wolb^+^ and fitted to the entire dataset for obtaining the final set of parameter estimates (Table 1), which are interpreted and used in the dynamic models of *Wolbachia* invasion and dengue transmission.

### *Wolbachia* invasion model

The conditions for *Wolbachia* invasion are assessed using a model that describes the population dynamics of two interbreeding *Ae. aegypti* populations: a resident *Wolbachia*-free population, *U*, and an introduced *Wolbachia*-carrier population, *W*. *Wolbachia* is considered to modify the longevity of its carriers by a factor *s*_l_, the fecundity by a factor *s*_f_, and to reduce the reproduction of *U* individuals via cytoplasmic incompatibility by a factor *s*_h_. Moreover, *Wolbachia* is assumed to modify the general susceptibility to mosquito pathogens by a factor that is positively correlated with that estimated for dengue viruses. The population dynamics is thus given by the following system of differential equations:

4 $$\frac{{{\rm d}u(x)}}{{{\rm d}t}} = q_u\left( x \right)aU\left( {\frac{{U + W\left( {1 - s_{\rm h}} \right)}}{M}} \right) - \left( {b + kM} \right)u\left( x \right) - x\lambda _Mu\left( x \right),$$

5 $$\frac{{{\rm d}w(x)}}{{{\rm d}t}} = q_w\left( x \right)aW\left( {1 - s_{\rm f}} \right) - \left( {\frac{b}{{1 - s_{\rm l}}} + kM} \right)w\left( x \right) - x\lambda _Mw\left( x \right),$$

where $q_{u}(x)$ and $q_{w}(x)$ are the susceptibility distributions of populations *U* and *W*, respectively, $U = \mathop {\int }\limits_0^\infty u(x){\rm d}x$ and $W = \mathop {\int }\limits_0^\infty w(x){\rm d}x$ represent the total Wolb^−^ and Wolb^+^ populations and *M* = *U* + *W* the total mosquito population. For parameter definitions and values, see Table 2.

This system of differential equations has been modified from previous studies^9,11^, and maintains the essential qualitative properties. It has three non-trivial equilibrium solutions: two stable and one unstable. The stable equilibria are referred to as the pre- and post-invasion equilibria, where either the *U* or the *W* population, respectively, has reached fixation. A third equilibrium can be found which accommodates both populations. Being unstable, however, any deviation will lead to fixation of one or the other population. Therefore, in practice, such a state does not persist, but instead sets a threshold frequency of *Wolbachia*-carriers necessary for invasion^19^—the invasion threshold, $\hat p$. Spatial dimensions may be included in the model and are expected to slow down local invasions due to reintroductions of non-carriers from neighbouring areas^23^. However, this is beyond the scope of this study.

Numerical solutions of the model were obtained in Matlab by discretizing each susceptibility distribution into 100 parts and simulating the resulting system of 200 ordinary differential equations.

### Dengue transmission model

To assess the effectiveness of *Wolbachia* in modifying the vectorial capacity of *Ae. aegypti*, we build a 4-serotype susceptible-infected-recovered (SIR) model for the human population, with transmission between humans mediated by mosquitoes that may carry or not the *Wolbachia* symbiont. Denoting by ${\cal V} = \left\{ {1,2,3,4} \right\}$ the set of dengue serotypes, the model for a homogeneous host population is formulated in set notation^38^ as

6 $$\frac{{{\rm d}S_\emptyset }}{{{\rm d}t}} = \mu H - \frac{{\beta \left( {\rho _U\mathop {\sum }\nolimits_{i \in {\cal V}} U_{Ii} + \rho _W\mathop {\sum }\nolimits_{i \in {\cal V}} W_{Ii}} \right)}}{H}S_\emptyset - \mu S_\emptyset,$$

7 $$\frac{{{\rm d}S_{\cal J}}}{{{\rm d}t}} = \varphi R_{\cal J} - \frac{{\beta \left( {\rho _U\mathop {\sum }\nolimits_{i \in {\cal V}{\mathrm{\backslash }}{\cal J}} U_{Ii} + \rho _W\mathop {\sum }\nolimits_{i \in {\cal V}{\mathrm{\backslash }}{\cal J}} W_{Ii}} \right)}}{H}S_{\cal J} - \mu S_{\cal J},\;{\mathrm{for}}\;\emptyset \ne {\cal J} \subseteq {\cal V},$$

8 $$\frac{{{\rm d}I_{{\cal J},i}}}{{{\rm d}t}} = \frac{{\beta \left( {\rho _UU_{Ii} + \rho _WW_{Ii}} \right)}}{H}S_{\cal J} - \left( {\gamma + \mu } \right)I_{{\cal J},i},\;{\mathrm{for}}\;{\cal J} \subset {\cal V},\;i \in {\cal V} \setminus {\cal J},$$

9 $$\frac{{{\rm d}R_{\cal J}}}{{{\rm d}t}} = \gamma \mathop {\sum }\limits_{i \in {\cal J}} I_{{\cal J} \setminus i,i} - \left( {\varphi + \mu } \right)I_i,\;{\mathrm{for}}\;\emptyset \ne {\cal J} \subseteq {\cal V},$$

and

10 $$\frac{{{\rm d}u_S(x)}}{{{\rm d}t}} = q_u\left( x \right)aU\left( {\frac{{U + W\left( {1 - s_{\rm h}} \right)}}{M}} \right) - \left( {b + kM} \right)u_S\left( x \right) - x\lambda _Mu_S\left( x \right) - x\mathop {\sum }\limits_{i \in {\cal V}} \lambda _{Hi}u_S\left( x \right),$$

11 $$\frac{{{\rm d}u_{Ii}(x)}}{{{\rm d}t}} = x\lambda _{Hi}u_S\left( x \right) - \left( {b + kM} \right)u_{Ii}\left( x \right) - x\lambda _Mu_{Ii}\left( x \right),\,{\mathrm{for}}\,i \in {\cal V},$$

12 $$\frac{{{\rm d}w_S(x)}}{{{\rm d}t}} = q_w\left( x \right)aW\left( {1 - s_{\rm f}} \right) - \left( {\frac{b}{{1 - s_{\rm l}}} + kM} \right)w_S\left( x \right) - x\lambda _Mw_S\left( x \right) - x\mathop {\sum }\limits_{i \in {\cal V}} \lambda _{Hi}w_S\left( x \right),$$

13 $$\frac{{{\rm d}w_{Ii}(x)}}{{{\rm d}t}} = x\lambda _{Hi}w_S\left( x \right) - \left( {\frac{b}{{1 - s_{\rm l}}} + kM} \right)w_{Ii}\left( x \right) - x\lambda _Mw_{Ii}\left( x \right),\;{\mathrm{for}}\;i \in {\cal V},$$

where $\lambda _{Hi} = \beta \mathop {\sum }\limits_{{\cal J} \subset {\cal V} \setminus i} I_{{\cal J},i}/H$, for *i* = 1,2,3,4, represent the force of infection of each serotype upon mosquitoes, *M* = *U* + *W* is the mosquito density, given that $U = \mathop {\int }\limits_0^\infty u(x){\rm d}x$, for $u\left( x \right) = u_S\left( x \right) + u_{I1}(x) + u_{I2}(x) + u_{I3}(x) + u_{I4}(x)$, and $W = \mathop {\int }\limits_0^\infty w(x){\rm d}x$, for $w\left( x \right) = w_S\left( x \right) + w_{I1}(x) + w_{I2}(x) + w_{I3}(x) + w_{I4}(x)$, and *H* represents the density of human host. Parameter definitions and values are in Table 2.

Heterogeneity in the risk of human hosts to acquire dengue infection is then introduced as in previous studies^39^. Specifically, human hosts are segmented into two risk groups, according to the degree of exposure to mosquito bites, such that the mean exposure is as in the homogeneous model to allow comparison, and the variance-to-mean ratio is a parameter initially set at 4 and varied for sensitivity analysis.

The total dimension of the complete system is 1124, consisting of the following: 62 histories of dengue infection in humans, times 2 to account for high and low-risk groups (124); 100 susceptibility classes of *Wolbachia*-free mosquitoes, times 5 to account for infection by each of the 4 dengue serotypes or none (500); 100 susceptibility classes of *Wolbachia*-carrying mosquitoes, times 5 to account for infection by each of the 4 dengue serotypes or none (500). All numerical solutions were obtained in Matlab.

In the absence of heterogeneity in human exposure or mosquito susceptibility, and without selection by mosquito pathogens (*λ*_M_ = 0), the type reproduction number^40^, which we denote here by *R*_0_, would be relatively straightforward to calculate. One infected human would lead to $\beta M/HH_{{\rm recovery} + {\rm death}}$ infected vectors, and one infected vector would lead to $\rho \beta /M_{{\mathrm{death}}}$ infected humans, where *ρ* is the proportion of infected mosquitos who are infectious, $H_{{\rm recovery} + {\rm death}}$ is the rate at which infectious humans stop transmitting due to recovery or death, and $M_{{\mathrm{death}}}$ is the rate at which mosquitos stop transmitting due to death. The number of human infections generated by one infected human would then be the product of these two numbers. With mosquito heterogeneity, the quantity becomes

14 $$R_0 = \frac{{\left( {\beta \mathop {\int }\nolimits_0^\infty xu\left( x \right){\rm d}x} \right)\left( {\rho _U\beta } \right)}}{{H\left( {\gamma + \mu } \right)\left( {b + kU} \right)}},$$

or

15 $$R_0 = \frac{{\left( {\beta \mathop {\int }\nolimits_0^\infty xw\left( x \right){\rm d}x} \right)\left( {\rho _W\beta } \right)}}{{H\left( {\gamma + \mu } \right)\left( {\frac{b}{{1 - s_{\rm l}}} + kW} \right)}},$$

depending on the *Wolbachia* carriage state of the mosquito population. When the two mosquito populations co-exist and mosquito pathogens are accounted for, the expression needs rearrangement to accommodate the differential death rates

16 $$R_0 = \frac{{\beta ^2}}{{H\left( {\gamma + \mu } \right)}}\left( {\rho _U\mathop {\int }\limits_0^\infty \frac{{xu\left( x \right)}}{{b + kM + \lambda _Mx}}{\rm d}x + \rho _W\mathop {\int }\limits_0^\infty \frac{{xw\left( x \right)}}{{\frac{b}{{1 - s_{\rm l}}} + kM + \lambda _Mx}}{\rm d}x} \right).$$

Including heterogeneity in human exposure to mosquito bites requires the extra factor ⟨*κ*^2^/*κ*⟩, where ⟨*κ*^2^⟩ is the second moment of the distribution of human exposure and the first moment (or mean) is ⟨*κ*⟩ = 1.

To assess the reduction in *R*_0_ attributed to the replacement of resident mosquitoes by those carrying *Wolbachia*, we calculate the ratio

17 $$\frac{{R_{0W}}}{{R_{0U}}} = \frac{{\rho _W\mathop {\int }\nolimits_0^\infty \frac{{xw\left( x \right)}}{{\frac{b}{{1 - s_{\rm l}}} + kW + \lambda _Mx}}{\rm d}x}}{{\rho _U\mathop {\int }\nolimits_0^\infty \frac{{xu\left( x \right)}}{{b + kM + \lambda _Mx}}{\rm d}x}},$$

with *w* and *u* determined by solving the model with and without *Wolbachia*, respectively, for the same transmission coefficient, *β*.

### Dengue incidence data

Dengue incidence was approximated from case notifications in Rio de Janeiro, Brazil (2010–2013) and Ho Chi Minh City, Vietnam (National Institute of Hygiene and Epidemiology (1995–2010) and Project Tycho, www.tycho.pitt.edu/dev/pnas/index.php). Based on official population counts, we adopted the approximate values of 6 million for Rio de Janeiro (Census Bureau, Brazilian Institute of Geography and Statistics (2010–2013) and 8 million for Ho Chi Minh City (General Statistics Office of Vietnam (1995–2010)).

### Model calibration

Model calibration was performed for Rio de Janeiro, Brazil, and Ho Chi Minh City, Vietnam, by calculating the endemic steady state as a function of the transmission coefficient *β* and adjusting this parameter to the annual incidence averaged over a period of 4 years (2010–2013 for Rio de Janeiro; 2007–2010 for Ho Chi Minh City). An expansion factor of 5 was applied to the official notifications to account for unapparent cases, as recommended by published studies in both countries^41,42^. Supplementary Fig. 1 shows the coexistence equilibrium solutions of the 4-serotype model, compared with lower dimensional versions generated with less serotypes (3, 2 or 1). The obtained incidence levels for Rio de Janeiro and Ho Chi Minh City are also plotted, showing, among other things, the need to adopt the full 4-serotype model to meet incidences as high as those in Brazil.

### Time series model

The model described was simulated to generate time series prior to *Wolbachia* introduction, to serve as a basis for projections of expected impact after releases occur. Simulations were performed with seasonal forcing on the transmission coefficient:

18 $$\beta \left( t \right) = \beta _0\left[ {1 - B\,{\rm cos} \left( {\frac{{2\pi \,t}}{{365}}} \right)} \right],$$

where *β*_0_ is the average transmission coefficient estimated for each scenario by the steady-state calibration above, and *B* is the amplitude of seasonal forcing on transmissibility, as well as on mosquito birth rates:

19 $$a\left( t \right) = a_0\left[ {1 - A\,{\rm cos} \left( {\frac{{2\pi \,t}}{{365}}} \right)} \right],$$

where *a* is the birth rate as in Table 2 and *A* is the amplitude of seasonal forcing on birth rate.

### Data availability

All data analysed in this paper are available from the cited publications Ferguson et al.^4^ and Souto-Maior et al.^14^.

## Electronic supplementary material

Supplementary Information
