# Supplementary material for: Variation in Wolbachia effects on Aedes mosquitoes as a determinant of invasiveness and vectorial capacity
Source: Nat Commun. 2018 Apr 16;9:1483. doi: 10.1038/s41467-018-03981-8 (PMC5902584; doi:10.1038/s41467-018-03981-8)
Supplement: Supplementary file 1 — Supplementary Information [file 41467_2018_3981_MOESM1_ESM.pdf]

Variation in *Wolbachia* effects on *Aedes* mosquitoes as a determinant of invasiveness and vectorial capacity

Supplementary Information
Figures 1-3
Tables 1-2

King at al.

Correspondence and requests for materials should be addresses to M.G.M.G. (email: gabriela.gomes@lstmed.ac.uk).

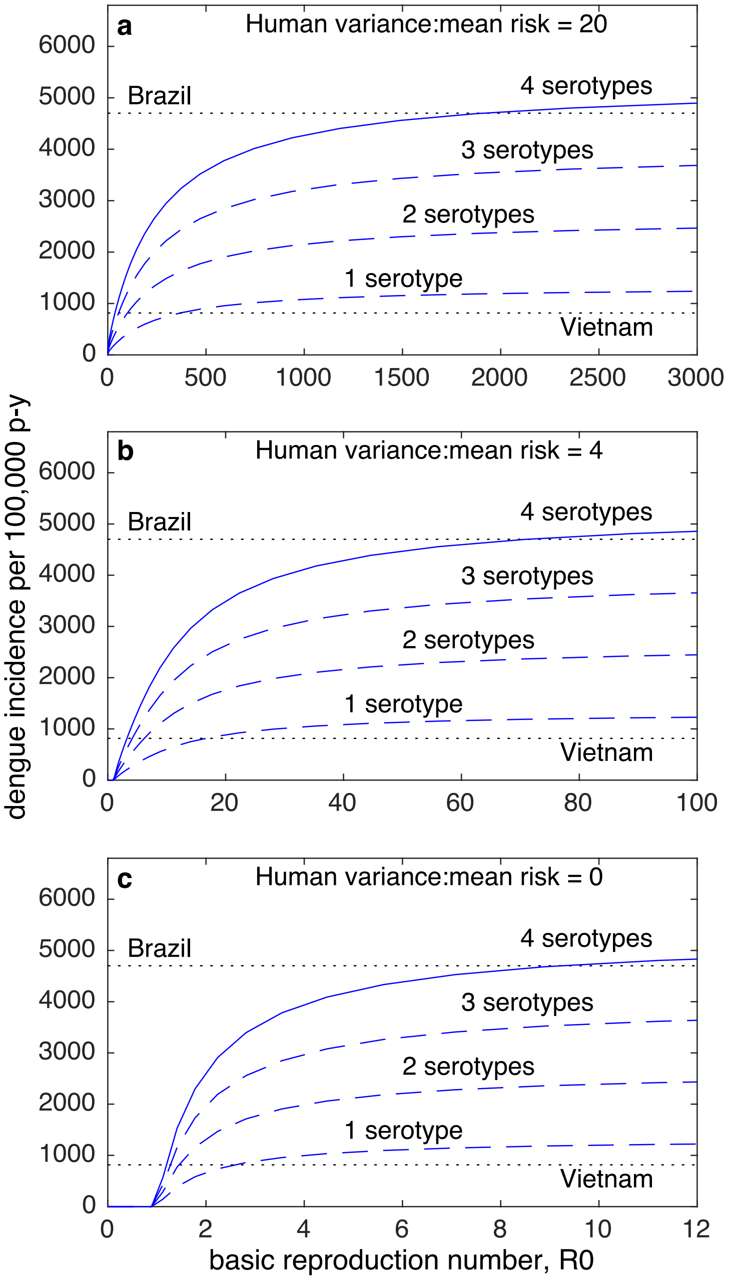

Supplementary Fig. 1 | Endemic equilibria for alternative numbers of circulating dengue serotypes. Blue curves are equilibrium solutions of models that differ in the number of circulating serotypes and are otherwise identical. Three scenarios are considered regarding the risk distribution in the human population: (a) heterogeneous, with a variance-to-mean ratio of 20; (b) heterogeneous, with a variance-to-mean ratio of 4; (c) homogeneous. Dotted lines mark dengue incidence in Rio de Janeiro, and Ho Chi Minh City, averaged over a 4-year period and scaled by an expansion factor of 5.

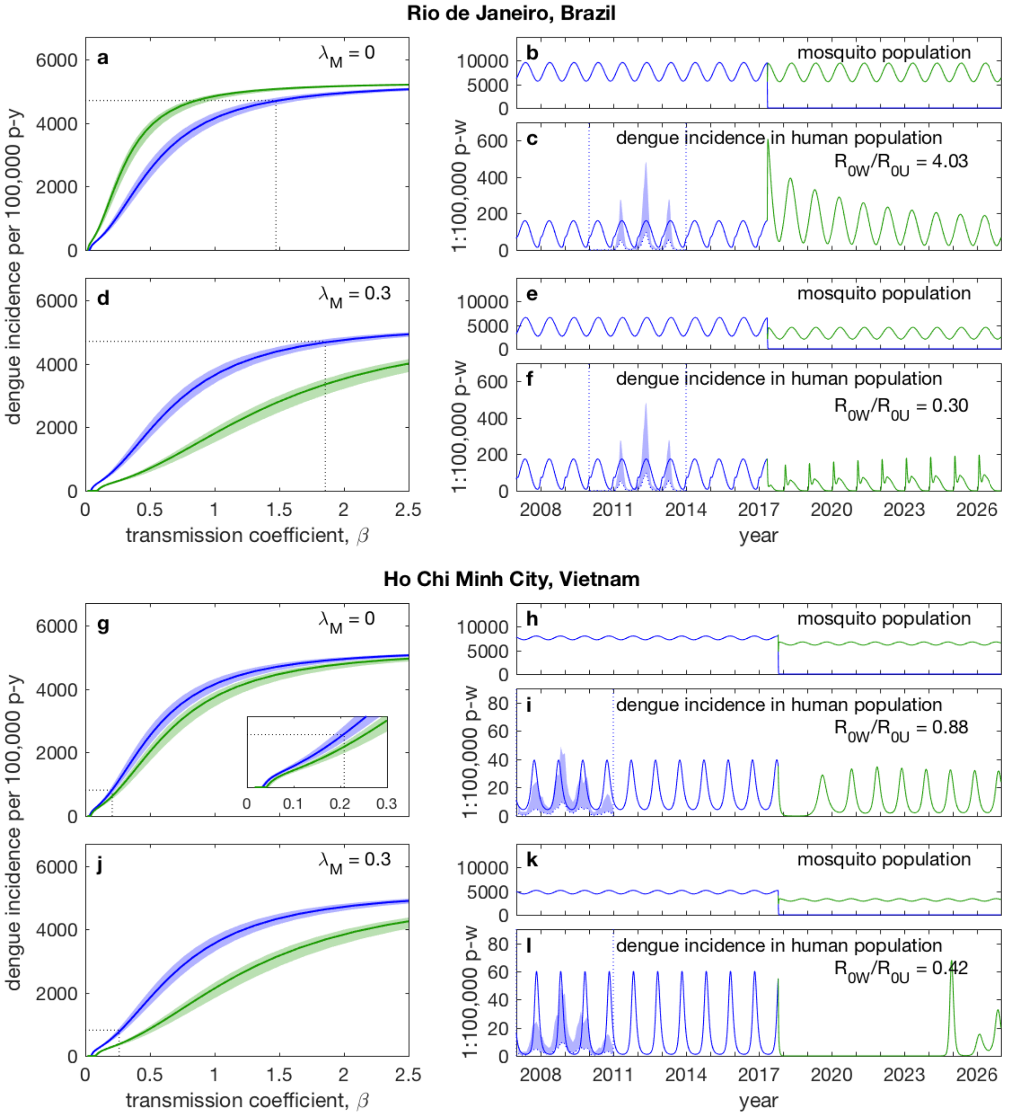

Supplementary Fig. 2 | Projected impact of *Wolbachia* on dengue transmission in human populations with heterogeneous risk. Colors indicate whether transmission is by *Wolbachia*-free (Wolb$^-$, in blue) or *Wolbachia*-carrier (Wolb$^+$, in green) mosquitoes. Left panels show dengue incidence versus transmission coefficient plotted from equilibrium solutions of a 4-serytope dengue model (Methods) on human populations with heterogeneous risk (variance to mean ratio of 20): (a,d) mosquitoes parameterized from experiments in Brazil and released under different forces of selection by natural pathogens: (a) $\lambda_M = 0$; (d) $\lambda_M = 0.3$. (g,j) same as (a,d) for Vietnam. Equilibrium curves adopted mean proportions of infected mosquitoes with detectable virus in the salivary glands[3], and shaded areas represent lower and upper bounds encountered when mosquitoes were stratified by challenge dose. Dotted lines mark dengue incidence in Rio de Janeiro, and Ho Chi Minh City, averaged over a 4-year period and scaled by an expansion factor of 5. Right panels show model simulations of *Wolbachia* releases taking place in 2017, starting from the conditions on the respective left panels: (b,e,h,k) mosquito population sizes; (c,f,i,l) dengue epidemics in humans (shaded areas refer to notification data; lower bound showing the original data and upper bound the data multiplied by an expansion factor of 5). Parameter values: $\beta = 1.47$ (c), $\beta = 1.86$ (f), $\beta = 0.21$ (i), $\beta = 0.26$ (l); $A = 0.25$, $B = 0.75$ (b,c,e,f); $A = 0.05$, $B = 0.07$ (h,i,k,l).

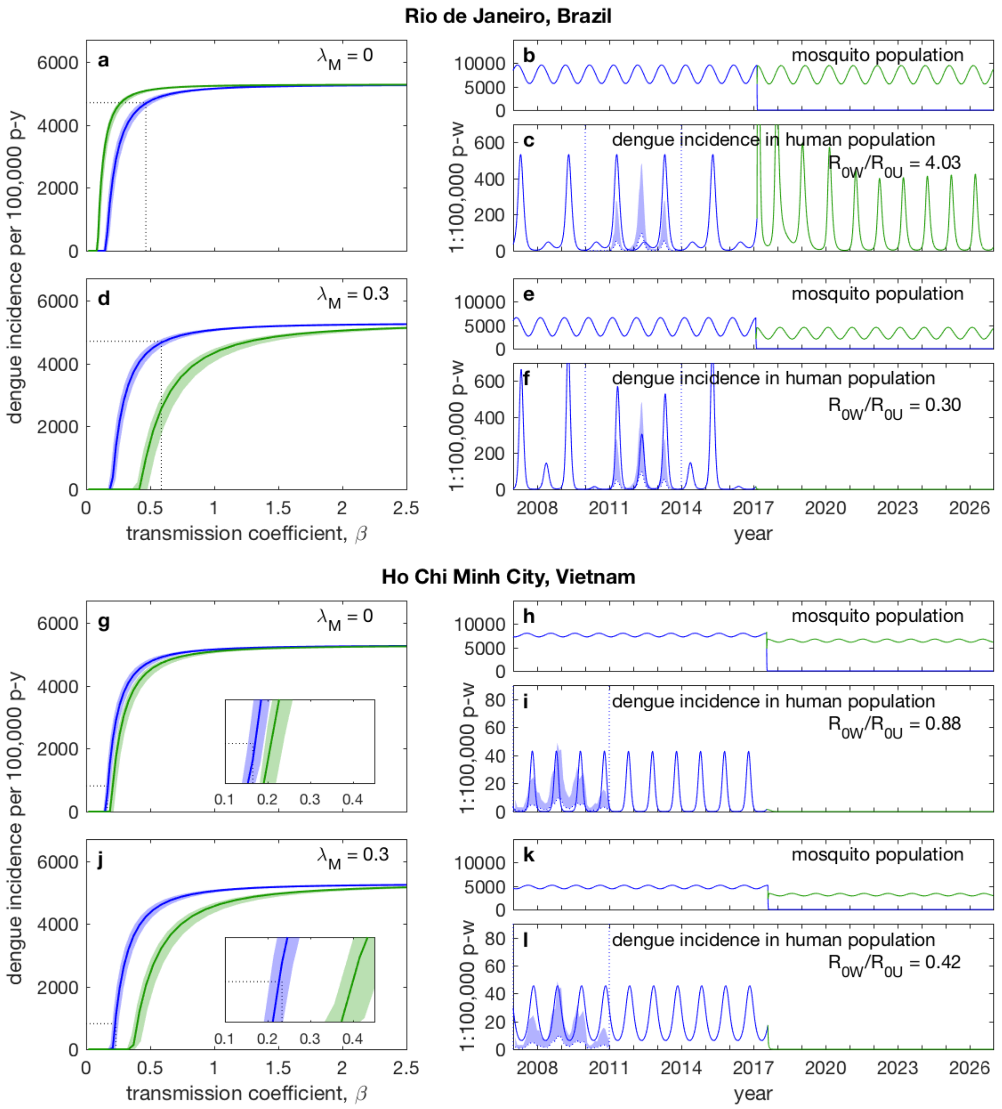

Supplementary Fig. 3 | Projected impact of *Wolbachia* on dengue transmission in human populations with homogeneous risk. Colors indicate whether transmission is by *Wolbachia*-free (Wolb^-, in blue) or *Wolbachia*-carrier (Wolb^+, in green) mosquitoes. Left panels show dengue incidence versus transmission coefficient plotted from equilibrium solutions of a 4-serytope dengue model (Methods) on human populations with homogeneous risk: (a,d) mosquitoes parameterized from experiments in Brazil and released under different forces of selection by natural pathogens: (a) $\lambda_M = 0$; (d) $\lambda_M = 0.3$. (g,j) same as (a,d) for Vietnam. Equilibrium curves adopted mean proportions of infected mosquitoes with detectable virus in the salivary glands[3], and shaded areas represent lower and upper bounds encountered when mosquitoes were stratified by challenge dose. Dotted lines mark dengue incidence in Rio de Janeiro, and Ho Chi Minh City, averaged over a 4-year period and scaled by an expansion factor of 5. Right panels show model simulations of *Wolbachia* releases taking place in 2017, starting from the conditions on the respective left panels: (b,e,h,k) mosquito population sizes; (c,f,i,l) dengue epidemics in humans (shaded areas refer to notification data; lower bound showing the original data and upper bound the data multiplied by an expansion factor of 5). Parameter values: $\beta = 0.47$ (c), $\beta = 0.59$ (f), $\beta = 0.17$ (i), $\beta = 0.23$ (l); $A = 0.25$, $B = 0.15$ (b,c,e,f); $A = 0.05$, $B = 0.45$ (h,i); $A = 0.05$, $B = 0.1$ (k,l).

Supplementary Table 1 | Parameter estimates from dose-response model fitting to experimental data. DIC denotes the Deviance Information Criterion. p denotes the infectivity of a viral challenge unit (50% tissue culture infective dose in Brazil, and $\log_{10}$ viral titre in Vietnam) to an average host.

Brazil			
	Homogeneous Wolb$^-$	Heterogeneous Wolb$^-$ ($p_{aux} = 1$)	
Parameter	p	α_{Wolb^-}	θ_{Wolb^-}
Median	1.051×10^{-7}	11.41	1.043×10^{-8}
95% CI	5.675×10^{-8}	1.128	1.214×10^{-9}
	1.838×10^{-7}	85.75	1.761×10^{-7}
DIC	7.424	10.67	

Vietnam			
	Homogeneous Wolb$^-$	Heterogeneous Wolb$^-$ ($p_{aux} = 1$)	
Parameter	p	α_{Wolb^-}	θ_{Wolb^-}
Median	3.599×10^{-8}	0.3640	4.112×10^{-6}
95% CI	3.315×10^{-8}	0.3041	2.364×10^{-6}
	3.740×10^{-8}	0.4344	7.569×10^{-6}
DIC	1039	105.2	

Supplementary Table 2 | Parameter estimates from dose-response model fitting to experimental data. DIC denotes the Deviance Information Criterion. p denotes the infectivity of a viral challenge unit (50% tissue culture infective dose in Brazil, and $\log_{10}$ viral titre in Vietnam) to an average host.

Brazil			
	Homogeneous Wolb^{-}	Heterogeneous Wolb^{-} ($p_{aux} = 10^{-8}$)	
Parameter	p	$\alpha_{Wolb^{-}}$	$\theta_{Wolb^{-}}$
Median	1.061×10^{-7}	2.487×10^{4}	4.265×10^{-4}
95% CI	5.675×10^{-8}	2.284	1.590×10^{-7}
	1.837×10^{-7}	6.572×10^{7}	6.205
DIC	7.424	7.570	

Vietnam			
	Homogeneous Wolb^{-}	Heterogeneous Wolb^{-} ($p_{aux} = 10^{-8}$)	
Parameter	p	$\alpha_{Wolb^{-}}$	$\theta_{Wolb^{-}}$
Median	3.625×10^{-8}	0.3643	407.0
95% CI	3.315×10^{-8}	0.3045	235.3
	3.740×10^{-8}	0.4349	755.7
DIC	1039	105.4	
